# Histomorphological evaluation, cell proliferation and endothelial immunostaining in oral and maxillofacial myofibroblastic lesions

**DOI:** 10.4317/medoral.25326

**Published:** 2022-10-16

**Authors:** Alina Gabriela Hassaf-Arreola, Claudia Haydee S Caro-Sánchez, Hugo Domínguez-Malagón, Maria Esther Irigoyen-Camacho, Oslei Paes de Almeida, Celeste Sánchez-Romero, Adalberto Mosqueda-Taylor

**Affiliations:** 1 Health Care Department, Metropolitan Autonomous University, Xochimilco, Mexico City, Mexico; 2 Department of Pathology, National Cancer Institute, Mexico City, Mexico; 3 Oral Pathology Section, Department of Oral Diagnosis, Piracicaba Dental School, University of Campinas, Brazil; 4 Department of Research, School of Dentistry, Universidad Juárez del Estado de Durango, Durango, Mexico

## Abstract

**Background:**

Myofibroblasts (MF) are mesenchymal cells with features of both fibroblasts and smooth muscle cells. Although these are usually reactive cells, they can lead to myofibroblastic tumors that may share clinical and histomorphological characteristics but with different prognosis. The aim of this study is to perform a histomorphological evaluation as well as to compare and evaluate two different cell proliferation immunomarkers and two endothelial markers in a group of oral and maxillofacial myofibroblastic lesions (MFL).

**Material and Methods:**

Cross-sectional and retrospective study. Demographic, clinical, histomorphological and immunohistochemical characteristics of 39 cases of MFL were analyzed. Immunohistochemical reactions were performed with the Ki67, MCM2, CD34 and CD105 antibodies. Kruskal-Wallis test and Spearman correlation analysis were used.

**Results:**

Four cases of nodular fasciitis (NF), 18 myofibromas (My), 6 desmoplastic fibromas (DF), 7 inflammatory myofibroblastic tumors (IMT) and 4 myofibroblastic sarcomas (MFS) were studied. There were twenty women (51.2%); the median age was 13 [Q1-Q3: 8-24] years and most cases occurred in the mandible (48.7%). A statistically significant difference with MCM2 immunostaining (*p*=0.0221) was observed between the MFL; furthermore, a correlation between CD34 and CD105 immunostaining in NF (*p* <0.0001) and IMT (*p*=0.0408), between MCM2 and CD34 in IMT (*p*=0.0362) and between MCM2 and CD105 in MFS (*p* <0001) were found.

**Conclusions:**

MCM2 immunostaining could assess more clearly the cell growth fraction in MFL. The correlation between MCM2 and CD34 in IMT and between MCM2 and CD105 in MFS are indicative of the high activity of these lesions. These results emphasize the importance of the studied immunohistochemistry markers as possible tools for a better characterization of some of the MFL.

** Key words:**Nodular fasciitis, myofibroma, desmoplastic fibroma, inflammatory myofibroblastic tumor, myofibroblastic sarcoma.

## Introduction

Myofibroblasts (MF) are mesenchymal cells with characteristics of fibroblasts and smooth muscle cells that have a spindle, stellar or pleomorphic morphology. They are not found in normal tissue and were originally identified in granulation tissue (1), but they can also occur in tumors, either as part of the stroma in the form of desmoplastic and reparative reactions, or as neoplastic components (2). Although they have a diverse cellular origin, local resident fibroblasts appear to be the most common source (3), whose transformation towards the myofibroblastic phenotype is mediated by TGF β1 and mechanical microenviromet (4).

Myofibroblastic lesions (MFL) are mainly composed by cells showing a myofibroblastic phenotype with varying patterns of differentiation, arranged on a variably collagenized matrix (2,5). These include pseudotumoral reactive lesions in the form of nodular fasciitis (NF), benign tumors such as myofibroma (My) and desmoplastic fibroma (DF), intermediate tumors like inflammatory myofibroblastic tumor (IMT), and malignant tumors such as myofibroblastic sarcoma (MFS). In addition to MF, these lesions share other features including collagen synthesis, some similar histomorphological patterns present in at least some areas of each lesion (6) and certain degree of cellular plemomorphism, although their biological behavior and prognosis are different (7).

The heterogeneous morphology of MF, coupled with the low frequency and histomorphological similarity shared by MFL, make their diagnosis often difficult (8,9). The aim of this article is to present the results of a study that describe the histomorphologic features and evaluates cellular proliferation and endothelial immunohistochemical markers in a group of oral and maxillofacial MFL in order to explore whether these are associated with differences in their histomorphology and to know if they are useful for defining more accurately their diagnosis.

## Material and Methods

- Sample

Thirty-nine cases of oral and maxillofacial MFL diagnosed from January 2001 to December 2018 were retrieved from the archives of the Oral Pathology Laboratory of the Metropolitan Autonomous University-Xochimilco and a private service of Oral Pathology in Mexico City.

Tissue samples diagnosed with NF, My, DF, IMT and MFS were selected according to the definition proposed for each entity by Enzinger & Weiss (10) and by the 2013 World Health Organization (WHO) Classification of Tumours of Soft Tissue and Bone (11). In addition, all selected cases had previous α-SMA positive immunohistochemical staining that confirmed their myofibroblastic nature. However, since there is no specific immunohistochemical panel for each MFL, the diagnosis of each lesion was verified by three pathologists (AMT, CHSCS and HDM), who were unaware of the previous diagnosis of the slide. Interexaminer agreement was satisfactory, with a kappa value ≥ 0.87. In those cases with no concordance, diagnosis was made by consensus among the same three pathologists, considering the clinical and histomorphological characteristics of each case.

- Sociodemographic, clinical and histomorphological study

Sociodemographic and clinical data on sex, age (only available in 37 MFL and location of the lesions, classified as jaw, maxillary and soft tissues, were recorded.

Histomorphological data were collected according to the cellular pattern, which was classified as storiform, fascicular and mixed (cases with a mixture of patterns, without predominance of a specific one, which also included the nodular pattern frequently described in myofibroma); the matrix was classified as dense, myxoid and mixed (composed of areas of dense collagen interspersed with myxoid areas); cellular pleomorphism was evaluated based on the morphology of the MF, which can be fusiform, stellar or epithelioid, so it was classified as "pleomorphic” when the lesion presented MF with variations in shape and/or size (pleomorphic) and as “spindle” when the myofibroblast cells showed only or predominantly spindle morphology. To evaluate vascularity, "active/immature vessels" were considered as those vessels with prominent endothelial cells and vessels with immature morphology, that is, with irregular shapes, tortuous, dilated, without defined endothelium, and distributed in a disorganized manner, and among these were those of hemangiopericytoid appearance, and "quiescent/mature vessels" were those which presented a structure composed of uniform endothelial cells, surrounded by mature connective tissue. Another evaluated variable was the infiltration to adjacent structures. Mitoses were evaluated in 10 high-power fields (HPF), starting from the most cellular and representative area of the lesion, from where 10 fields in 400x were selected by moving horizontally or vertically around that area, while the necrosis evaluation was performed on the entire sample, excluding ulcerated areas.

- Immunohistochemical study

Three μm thick sections were obtained from 10% formalin fixed tissues included in paraffin blocks. Those employed for Ki67 (monoclonal mouse, DAKO®, 1:100), CD34 (monoclonal mouse, DAKO®, 1:50) and CD105 (monoclonal mouse, DAKO®, 1:50) immunomarkers were mounted on silanized slides, while in the case of MCM2 (monoclonal rabbit, Bio SB®, 1:150) they were mounted on electro-charged slides. All were subjected to the immunoperoxidase method.

Only nuclear staining was considered positive reaction in cell proliferation evaluation (Ki67 and MCM2). The immunostaining percent was calculated by hot spot (higher number of neoplastic cells/higher immunostaining) at 100x with the Ki67 antibody in a representative region according to the method proposed by Jang *et al*. (12). The first field at 400x was chosen in the selected area, starting in the portion of the left periphery, and subsequently four adjacent fields were selected by moving horizontally to the right of the first selected field. Photomicrographs were taken and stored in jpg files (IMAGE-J 1.45), and in each of them a 6x6 graticule was placed and cell (MF) counting was performed manually. MF were identified based on their morphological characteristics, from fusiform cells to pleomorphic cells; no inflammatory cells or endothelial cells with a positive reaction were included. The count started in the upper-left corner and ended in the upper-right corner. The immunostaining percent was calculated dividing the amount of MF with positive nuclear reaction by the total MF in the five fields and multiplied by one hundred (13).

For endothelial markers analysis (CD34 and CD105) a Weidner *et al*. (14) method modification was used as detailed below. Only when there was membrane and cytoplasmic positive reaction of endothelial cells of vessels with lumen was considered as positive. A representative area (according to the histomorphological criteria of each lesion) at 100x in H&E was selected (transferred to the samples with endothelial stains CD34/CD105), and in these areas the first field in 400x was chosen, starting in the portion of the left periphery, and subsequently four adjacent fields were selected by scrolling horizontally to the right of the first selected field. Photomicrographs of the representative fields were taken, which were stored in jpg files (IMAGE-J 1.45) and the manual counting of the vessels with lumen with positive reaction was performed. Individual cells or groups of cells with positive reaction, but without lumen were not considered as vascular elements to avoid counting elements that did not correspond to true vessels. The immunostaining average was calculated dividing the number of vessels with lumen with positive reaction by the total of studied fields.

- Statistical analysis

For statistical comparison between the immunostainings, Kruskal-Wallis test was used. For the correlation between the immunostainings and the studied MFL, Spearman's coefficient test (Spearman's ρ) was used. The level of statistical significance for the hypothesis tests was *p* < 0.05. Statistical analysis was performed with the JMP V12 package.

## Results

There were 4 cases of NF, 18 My, 6 DF, 7 IMT and 4 MFS. Demographic and clinical characteristics of the 39 patients with MFL are shown in Table 1, which shows that 51.2% of cases occurred in women, with a median age of 13 years (Q1-Q3:8-24). The mandible was the most frequent location (19/48.7%), followed by soft tissues (11/28.1%); however, a higher number of the MFS cases observed in women occurred in the maxilla (75%).

Histopathological features are summarized in Table 2, where it is seen that storiform and fascicular cellular patterns were the most common (15/38.4% respectively). Twenty-three lesions (58.9%) had mixed matrix (composed of dense collagen areas intermixed with myxoid areas), being My the lesion that most frequently presented this feature (61.1%) while most cases of DF had a dense collagen matrix (83.3%). Thirty-three MFL had some degree of cellular pleomorphism (84.6%), and MFS presented this finding more frequently and more intensely (4/100%), followed by IMT (85.7%). The active/immature vessels were the most common type of vasculature (69.2%), observed in all cases of MFS. On the other hand, DF presented a higher frequency of quiescent/mature vessels (83.3%). Infiltration into adjacent structures was observed in 23 MFL (58.9%), being this finding most evident in MFS (100%) and NF (75%), and less common in DF (33.3%). Between 1 and 5 mitoses in 10 HPF were found in 13 cases (33.3%), while atypical mitosis was only found in MFS, half of which (50%) showed 6 or more mitosis in 10 HPF. On the other hand, only 9 MFL had necrosis (23%), being IMT the one that presented it most frequently (71.4%).

- Immunohistochemical findings

**Figure 1:**
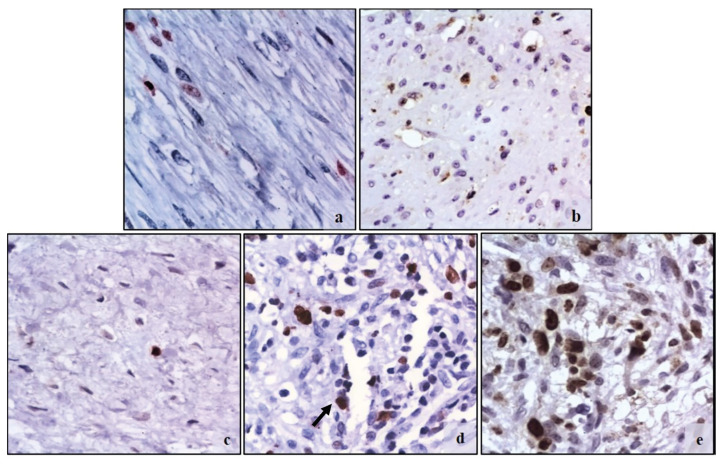
Ki67 immunostaining in MFL. a) Nodular fasciitis. Fusiform cell population with low reactivity. b) Myofibroma. Positively reacting MF scattered around immature-looking vessels, c) Desmoplastic fibroma. A MF with positive reaction, indicating the low proliferative capacity of this lesion, d) Inflammatory myofibroblastic tumor. Numerous reactive MF (it was avoided to count endothelial cells (arrow)) with positive reaction to Ki67, e) Myofibroblastic sarcoma. Large population of MF with positive reaction dispersed over a myxoid matrix (Ki67 400x).

**Figure 2:**
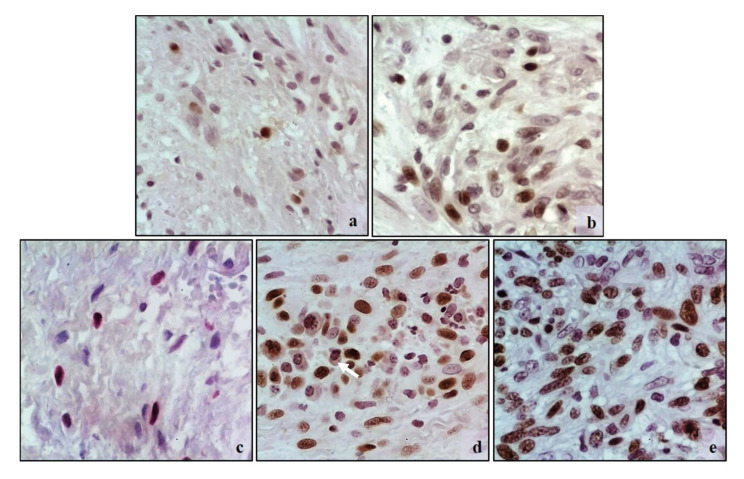
MCM2 immunostaining in MFL. a) Nodular fasciitis. Low reactivity in MF. b) Myofibroma. Abundant positive reaction MF dispersed in a myxoid matrix. c) Desmoplastic fibroma. Few positive MF indicating the low proliferative capacity of this MFL. d) Inflammatory myofibroblastic tumor. Population of MCM2 reactive MF, where only myofibroblastic cells were counted, (inflammatory cells, such as plasma cells were excluded (white arrow)). e) Myofibroblastic sarcoma. Abundant positive reaction MF. (MCM2 400x).

**Figure 3:**
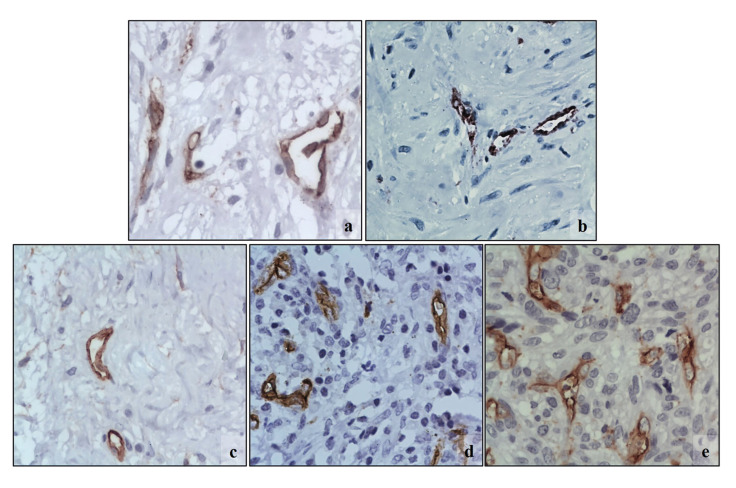
CD34 immunostaining in endothelial cells. a) Nodular fasciitis. CD34 positive vessels dispersed in a myxoid matrix and interspersed with the cell population. b) Myofibroma. Irregular vessels with positive reaction. c) Desmoplastic fibroma. Reactive vessels dispersed in a dense collagen matrix and sparse cell population d) Inflammatory myofibroblastic tumor. Immature-looking vessels with positive reaction, with irregular shapes and disorganized pattern interspersed with tumoral and inflammatory cells. e) Myofibroblastic sarcoma. Abundant capillaries scattered among tumor cells. (CD34 400x).

**Figure 4:**
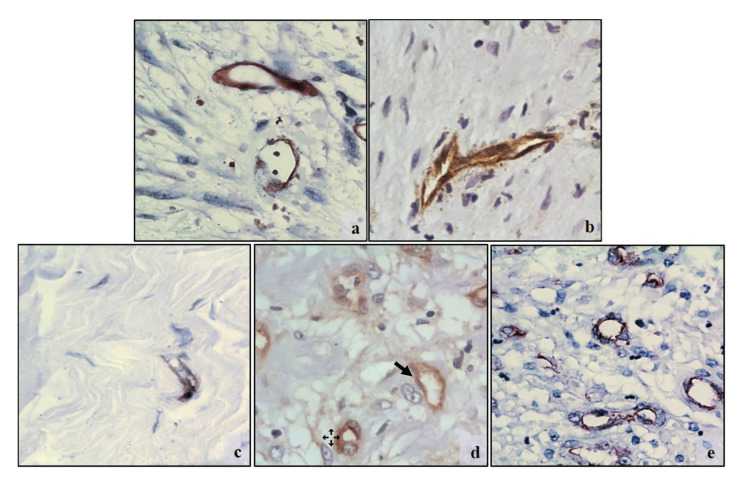
CD105 immunostaining in endothelial cells. a) Nodular fasciitis. Reactive vessels dispersed in a myxoid matrix, b) Myofibroma. Irregular vessel with positive reaction. c) Desmoplastic fibroma. Vessel in a dense collagen matrix and sparse cell population. d) Inflammatory myofibroblastic tumor. Positive reaction in active-looking vessel with prominent endothelial cells (*) and immature-looking vessel (arrow), interspersed with tumor myofibroblastic cells in a mixoid matrix. e) Myofibroblastic sarcoma. Multiple dispersed capillaries with positive reaction (CD105 400x).

Ki67 and MCM2 medians were higher in the MFS as compared to the rest of the MFL (43% [Q1-Q3:10.9-57] and 49.43% [Q1-Q3:15.4-64] respectively), but there was only statistically significant difference with the MCM2 marker (*p*=0.0221) (Table 3, Fig. 1, Fig. 2). CD34 median was very similar in all MFL (*p*=0.441), while CD105 median was higher (3.1 [Q1-Q3:1.3-5]) in SMF, but no statistically significant difference was found (*p*=0.247) (Table 3, Fig. 3, Fig. 4).

**Table 1:**
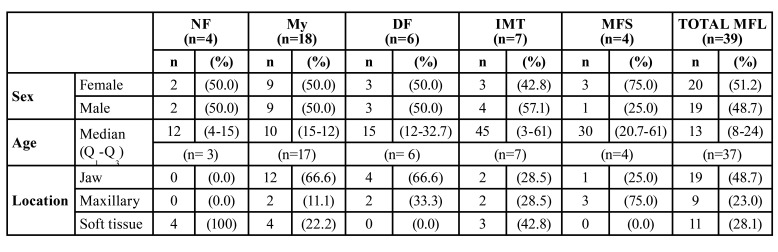
Demographic and clinical characteristics of 39 patients with MFL.

**Table 2:**
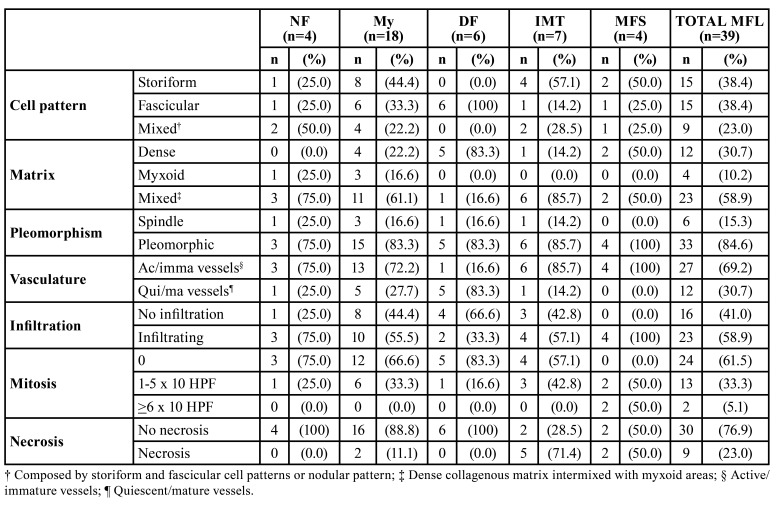
Histomorphological features of 39 MFL.

**Table 3:**
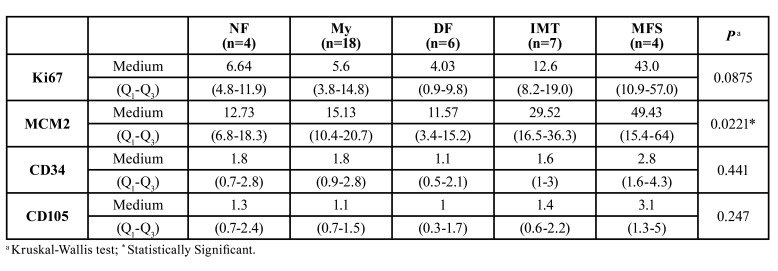
Median immunostaining of cell proliferation (Ki67 and MCM2) and endothelial markers (CD34 and CD105) in 39 MFL.

As shown in Table 4, endothelial markers (CD34 with CD105) correlation was observed in both, NF (ρ= 0.9021) (*p* <0.0001), and IMT (ρ= 0.8035) (*p*= 0.0408); as well as between MCM2 and CD34 in the IMT (ρ= 0.7746) (*p*= 0.0362) and between MCM2 and CD105 in the MFS (ρ= 0.8177), (*p* <0.0001).

**Table 4:**
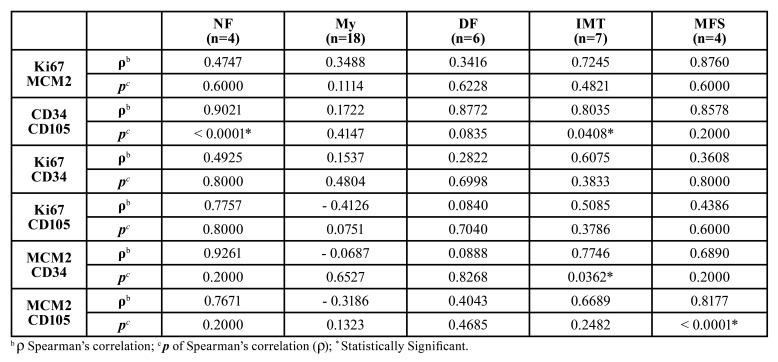
Correlation between the presence of cell proliferation (Ki67 and MCM2) and endothelial markers (CD34 and CD105) in 39 MFL.

## Discussion

In spite of being composed to a greater or lesser extent by MF, the poorly specific immunophenotype of these cells does not allow to establish clear differences among the diverse MFL, so diagnosis still depends mainly on their histomorphological pattern. Because these lesions tend to exhibit marked vascularity and, in some cases, high proliferation rate, we focused our analysis mainly in these elements, as these may be responsible for causing diagnostic confusion to differentiate between them.

According to the microscopic characteristics found in this study, storiform and fascicular were the most common cellular patterns in MFL (38.4% each, respectively), which coincides with what has been described for NF (15), IMT (16) and MFS (17). With respect to My, a review of cases by Vered *et al*. identified predominance of the nodular zonal pattern (described as storiform/fascicular in our study) in 41 of their cases (78%) (18), whereas we only observed it in 22.2% in our cases. These differences are difficult to explain, as there is no consensus about classification of histological patterns. In our study it was carried out according to the predominance of the pattern, even if other histological arrangements were present. All cases of DF exhibited a fascicular pattern, a finding that is consistent with previous reports (19,20), which suggests it is due to its higher collagen production and lower cell proliferation; therefore, the mixed patterns in NF, My and IMT imply these are lesions in a transition process by maturation stages, which explains the morphological variability reported.

More than a half of the MFL (58.9%) in this study had a mixed matrix [NF (75%), My (61.1%), IMT (85.7%) and MFS (50%)], which is similar to the findings reported by other authors (16,17,21-23). On the other hand, 83.3% of DF cases showed a dense collagen matrix, in accordance to what has been found in other studies (24) which probably relates to the predominant cell pattern (fascicular).

Most of the studied MFL (84.6%) showed some degree of cellular pleomorphism, a finding previously mentioned in some series of NF (25), My (7,18,26), DF (19), and MFS (17), which may possibly be due to the variable morphology a MF may present, which ranges from fusiform to epithelioid cells that often acquire a pleomorphic appearance.

Active/immature vessels were evident in a high percentage of the MFL included in this study [NF (75%), My (72.2%), IMT (85.7%) MFS (100%)], which also has been reported for My (7,21), IMT (16) and MFS (5). Regarding NF, it has been described that this lesion goes through different stages of development, so, at the beginning it presents vascular clefts without defined endothelium (immature vessels) and capillaries with prominent endothelial cells (active vessels) while in more advanced lesions vascularization is characterized by the presence of mature vessels lined by endothelial cells and surrounded by connective tissue (22,25), suggesting initial lesions are very active. DF cases, on the other hand, had mostly quiescent/mature vessels (83.3%), similar to those reported by Hauben *et al*. who studied 13 cases from the Netherlands, Switzerland and Belgium, which showed well-developed capillaries and arterioles in relation to lower cellularity and mature collagen (19).

Most MFL included in our study disclosed infiltration to adjacent structures, similar to what has been observed by other authors (15,26-28). However, only 33.3% of DF cases had this feature, which is lower than reported by Fisker and Philipsen [9/15 (60%) cases] (29); a possible explanation to this could be the fact that, as it is an intraosseous tumor that was evaluated in our study through incisional biopsies, it is not possible to rule out the presence of invasion in areas that could be only evident when studying the whole surgical specimen.

Thirteen MFL (33.3%) had between 1-5 mitosis per 10 HPF [My (33.3%), NF (25%), DF (16.6%), IMT (42.8%) MFS (50%)], similar to what has been previously reported in some MFL (21,25,27), except for DF, in which mitosis are uncommon (19), as seen in our study. Unsurprisingly, the presence of atypical mitoses occurred exclusively in MFS, half of which had 6 or more mitoses per 10 HPF. This finding is important because it has been proposed recently that the presence of 6 or more mitosis per 10 HPF in conjunction with necrosis (which were detected in half of the cases of MFS) is considered as diagnostic criteria of high-grade MFS (17). On the other hand, no cases of NF or DF in our study presented necrosis, similar to previously published data (19,24,25,29).

The cell proliferation index evaluated with the Ki67 antibody in MFL varied and corresponded with the histopathological characteristics and biological behavior of each lesion, being higher in MFS (43%) and lower in DF (4.03%); however, no statistically significant difference was identified. It has been proposed recently that MF in short-evolving NF (no more than two months), maintain a proliferative activity greater than 30%, which is higher than those found in NF of longer time of evolution (23). According to these observations, the results of this study suggest that our NF cases possibly correspond to long-evolving lesions, since the median Ki67 immunostaining was only 6.64%; however, as there are no clinical data about their evolution, we could not confirm it.

MCM2 belongs to a group of key proteins that start DNA replication (30), and in this study it was the only marker that presented a statistically significant difference (*P*=0.0221) between the diverse MFL and could therefore be considered as a more accurate marker to evaluate cell proliferation as compared to Ki67.

No statistically significant differences were found when evaluating vascularity through CD34 and CD105 (*p*= 0.441, *p*= 0.247 respectively). The low concordance between the active/immature vessels with CD105 immunoreaction is probably because the endoglin expression decreases to allow vessel stabilization and recruitment of wall cells (mature vessel) (31), which would explain why a proportion of immature vessels can be negative for CD105 and positive only for CD34.

The clinicopathological description and the immunohistochemical results allow us to assume the MFL have a common origin, but their cellular components are in different maturation stages and proliferative activity, where benign lesions with increased angiogenesis and cell proliferation (such as NF and My) represent lesions that may persist with their initial proliferative changes, while those with less immunoexpression (such as DF) would be lesions that have progressed to a more mature stage and therefore have the appearance of late-stage reparative reactions where fibrosis predominates; these findings are consistent with the development of the desmoplastic process, since some studies have suggested that an overtly fibrotic microenvironment does not promote angiogenesis (32).

In NF and IMT cases, a statistically significant correlation (*p* < 0.0001 and *p*= 0.0408 respectively) between the CD34 and CD105 endothelial markers was identified, which suggest that most vessels in these MFL are active and recently formed. In NF, these findings are likely to be related to its rapid growth while in IMT it could be due to the high proliferative ability induced by the myofibroblasts themselves, which secrete a variety of proangiogenic growth factors including vascular endothelial growth factor (VEGF), basic fibroblast growth factor (bFGF), transforming growth factor β (TGFβ), platelet-derived growth factors (PDGF), hepatocyte growth factor (HGF), connective tissue growth factor (CTGF) and interleukin-8 (IL-8) (32). This study also found a statistically significant correlation between MCM2 and CD34 in IMT (*p*= 0.0362), indicating that increasing cell proliferation would rise neovascularization and vice versa, a correlation that is consistent with its locally infiltrative neoplastic character, frequent recurrences, but low potential for metastasis.

A statistically significant correlation between MCM2 and CD105 immunostaining was identified in the MFS cases (*p*<0.0001), confirming that an increased cellular proliferation would increase vessel formation and vice versa, as observed in many other malignancies (32), and this could possibly be related to TGF β expression, which in turn would lead to myofibroblastic differentiation and interaction with Endoglin accessory protein (CD105) to trigger angiogenesis.

In conclusion, this study describes the histomorphologic features of oral and maxillofacial MFL and reveals the complexity of making definitive diagnosis due to the similarity of features among them. In addition, our results suggests that MCM2 may help to assess more clearly the cell growth fraction in MFL and this could be a useful tool to support the diagnosis of these lesions. The correlations between CD34 and CD105 in NF and IMT suggest a high neovascularization, probably related to the high proangiogenic activity of myofibroblasts, while the correlation between MCM2 and CD34 in IMT, and between MCM2 and CD105 in MFS, indicate the myofibroblasts pose high activity to proliferate and induce angiogenesis in these lesions. The information obtained in this work provides a better understanding of the salient features of MFL and confirms that histomorphology remains as one of the most important diagnostic tool for this particular group of entities, and provides new information about the usefulness of immunohistochemistry for a better definition of each of the well-recognized MFL.
